# Bilateral muscle fiber and nerve influences by TNF-alpha in response to unilateral muscle overuse – studies on TNF receptor expressions

**DOI:** 10.1186/s12891-017-1796-6

**Published:** 2017-11-28

**Authors:** Lina Renström, Per Stål, Yafeng Song, Sture Forsgren

**Affiliations:** 1 Department of Integrative Medical Biology, Section of Anatomy, Umeå University, Umeå, Sweden; 2 Perelman School of Medicine & Pennsylvania Muscle Institute, University of Pennsylvania, Philadelphia, PA USA

**Keywords:** TNF-alpha, Muscle, Myositis, TNFR1, TNFR2, Nerve structures

## Abstract

**Background:**

TNF-alpha is suggested to be involved in muscle damage and muscle inflammation (myositis). In order to evaluate whether TNF-alpha is involved in the myositis that occurs in response to muscle overuse, the aim was to examine the expression patterns of TNF receptors in this condition.

**Methods:**

A rabbit muscle overuse model leading to myositis in the soleus muscle was used. The expression patterns of the two TNF receptors Tumor Necrosis Factor Receptor type 1 (TNFR1) and Tumor Necrosis Factor Receptor type 2 (TNFR2) were investigated. In situ hybridization and immunofluorescence were utilized. Immunostainings for desmin, NK-1R and CD31 were made in parallel.

**Results:**

Immunoreactions (IR) for TNF receptors were clearly observed in white blood cells, fibroblasts and vessel walls, and most interestingly also in muscle fibers and nerve fascicles in the myositis muscles. There were very restricted reactions for these in the muscles of controls. The upregulation of TNF receptors was for all types of structures seen for both the experimental side and the contralateral nonexperimental side. TNF receptor expressing muscle fibers were present in myositis muscles. They can be related to attempts for reparation/regeneration, as evidenced from results of parallel stainings. Necrotic muscle fibers displayed TNFR1 mRNA and TNFR2 immunoreaction (IR) in the invading white blood cells. In myositis muscles, TNFR1 IR was observed in both axons and Schwann cells while TNFR2 IR was observed in Schwann cells. Such observations were very rarely made for control animals.

**Conclusions:**

The findings suggest that there is a pronounced involvement of TNF-alpha in the developing myositis process. Attempts for reparation of the muscle tissue seem to occur via both TNFR1 and TNFR2. As the myositis process also occurs in the nonexperimental side and as TNF receptors are confined to nerve fascicles bilaterally it can be asked whether TNF-alpha is involved in the spreading of the myositis process to the contralateral side via the nervous system. Taken together, the study shows that TNF-alpha is not only associated with the inflammation process but that both the muscular and nervous systems are affected and that this occurs both on experimental and nonexperimental sides.

## Background

Tumor necrosis factor alpha (TNF-alpha) is a cytokine that is involved in several biological processes, including activation of white blood cells, apoptosis, cell survival and cell proliferation [1]. It is expressed in several types of white blood cell, as well as other cell types, and is involved in recruitment and activation of immune cells [2]. TNF-alpha is signalling via the TNF Super Receptor Family. This is a family of several glycoprotein receptors in which Tumor Necrosis Factor Receptor type 1 (TNFR1) and Tumor Necrosis Factor Receptor type 2 (TNFR2) are the most studied.

TNF-alpha has been considered to possibly be an important factor in muscle inflammation/damage [3, 4]. Muscle inflammation (myositis) is a condition which can be caused by several inflammatory myopathies [5], in which the TNF-alpha system is involved [6, 7], as well by muscle stress [8]. Several studies have shown a marked role of TNF-alpha in such conditions [6, 9]. In a rabbit model leading to muscle inflammation due to overuse, we noted involvement of TNF-alpha in the myositis process [10]. That included findings showing that overuse of one of the extremities not only led to muscle inflammation and damage ipsilaterally, but also contralaterally, and that the TNF-alpha system was upregulated in both extremities. This suggests that the TNF-alpha system can have effect via the nervous system.

Muscle tissue is a tissue that can be markedly influenced by various factors (for a review, see [11]. That includes influences in the different stages that occur in muscle differentiation and muscle injury, not least effects elicited by the central nervous system, growth factors and endocrine-metabolic events [12]. The role of innervation has been especially emphasized, denervation eventually leading to muscle fiber death [13]. Physical exercise has also a great impact on skeletal muscle, resulting in an effect on TNF-alpha production [11]. A very marked exercise possibly leads to inflammation and damage of the muscle tissue [14]. There is also an effect of diet on muscle homeostasis [14, 15] and an effect of vibration stimulation on the musculoskeletal system [16].

The function of TNF-alpha in muscle tissue can be very heterogeneous. One possibility is that TNF-alpha may play an important role in the pathogenesis of the muscle destruction that occurs in myositis [17]. However, results of experimental observations implicate that TNF-alpha also has a direct action on the muscle cells in muscle regeneration [18]. The major role of TNF-alpha in inflammatory and non-inflammatory myopathies is on the whole suggested to be related to regeneration of the muscle fibers [19]. Nevertheless, TNF-alpha is involved in the development of injury following ischemia in various organs [20], but it is presumably also involved in wound repair [21].

The functional importance of TNF-alpha in the situation with myositis in response to muscle overuse is completely unclear. Thus, there is no information whether TNF receptors are expressed in the muscle fibers or the inflammatory cell infiltrates and the blood vessel walls. An important question is also whether TNF-alpha is influencing the peripheral innervation of the muscle. The non-existence of information concerning TNF receptor expression patterns for the peripheral innervation in muscle tissue is hampering the understanding of the presumable TNF-alpha effect on the nervous system. Here it should be recalled that TNF-alpha can be produced by neurons [22–25] and that TNFR1 as well as TNFR2 are shown to be expressed by neurons as well as glia cells [26, 27]. These expressions are reported to be increased in pathological situations, such as after nerve injury [28] and lumbar facet joint injury [29].

The purpose of this study was to give an understanding of the role of TNF-alpha in the muscle damage and muscle inflammation that occurs in response to marked overuse. To test the hypothesis that TNF-alpha is involved in such a condition, we used a rabbit muscle overuse model and investigated the presence of TNFR1 and TNFR2 in the myositis process. Via evaluating the expression patterns for the receptors, a conclusion concerning the role of TNF-alpha for the muscle fibers, the innervation and the inflammation could be obtained. Our study will deepen the knowledge of the importance of TNF-alpha in the processes that occur in the myositis process in response to marked muscle overuse.

## Methods

### Animals and experimental procedures

A total of 46 female rabbits were used in the studies. The animals, New Zealand rabbits, had an average weight of 4 kg and were 6–9 months old. The animals were divided into eight groups with five or six animals in each. Three groups were attending a muscle overusing experiment for 1, 3 or 6 weeks. One group did not participate in the muscle overuse experiment and is from now on considered as the control group. To emphasize the effects of an inflammatory stress on the muscle tissue, other animals enrolled in the experiment for 1 week were given injections of pro-inflammatory agents. See Table 1 for overview of the groups.

**Table 1:** The characteristics of the animal groups are shown. Substances given were sodium chloride (NaCl), substance P (SP), DL-Thiorphan (Th) and Captopril (Cap)

Group	Experiment	Injected	No. Animals
1	No	No	6
2	1 week	No	6
3	1 week	Yes: NaCl	5
4a	1 week	Yes: SP + Th + Cap	5
4b	1 week	Yes: Cap + Th	6
4c	1 week	Yes: Cap	6
5	3 weeks	No	6
6	6 weeks	No	6

The exercise experiment was performed when rabbits were anesthetized. Intramuscular injections of fentanyl-fluanisone (0.095 mg/kg fentanyl citrate and 3 mg/kg fluanisone) (VetaPharma) and diazepam (1 mg/kg) (Roche) were given at the start of the experiments. Fentanyl-fluanisone (0.03 mg/kg fentanyl citrate and 1 mg/kg fluanisone) was thereafter injected every 30–45 min to sustain anesthesia. We used a “kicking machine” that had been originally designed by Backman et al. [30]. Only one of the legs (the right leg) was attached to the machine. Hereby, the right foot, but not the left foot, was moving passively. Via the use of a pneumatic piston, repetitive passive flexion-extension of the right ankle was achieved. There was a band placed around the hip/pelvis to restrict ankle movements on the left side. By use of electrical stimulation induced via surface electrodes, an active contraction was furthermore induced during the plantar flexion phase. The surface electrodes (Pediatric electrodes 40 426A, Hewlett Packard, Andover, MA, USA) were placed 2 cm apart over the triceps surae on the right side. By the use of a microswitch, which triggered the stimulator unit (Disa stimulator Type 14E, Disa Elektronik A/S, Herlev, Denmark). The stimulation was synchronized with the plantar flexion movement of the piston. 85 ms after the initiation of plantar flexion an impulse of 0.2 ms duration was delivered at an amplitude of 35-50 V. The movement frequency of the right foot was 150 movements per minute. This exercise session lasted for in total 2 h and was repeated every second day for 1, 3 or 6 weeks. In response to the exercise, there was a marked overuse of the muscle. For further details see [10].

Injection treatment was given directly after the muscle experiment for animals subjected to 1 week training program (groups 3, 4a–c). The injections included injections with Captopril (Cap) (C4042, Sigma), Substance P (SP) (S6883, Sigma) and DL-Thiorphan (Th) (T6031, Sigma). The substances were given in different combinations (groups 4a–c) (Table 1). One group (group 3) was injected with sodium chloride (NaCl). Captopril is well-known to be an ACE inhibitor and DL-Thiorphan is a neutral endopeptidase inhibitor. The site of injection was the loose peritendinous tissue around the Achilles tendon of the experimental side.

For analgesia the rabbits were given buprenorphine (0.01–0.05 mg/kg) (Schering-Plough) subcutaneously after every training session. Between trainings, rabbits were kept in cages allowing movement.

One day after the last training session, the rabbits were euthanized with an excessive amount of sodium pentobarbital and the entire triceps surae muscles from both legs were excised. The soleus part of the muscles was further dissected out and cut into pieces. These pieces were either directly mounted in an OCT compound (Tissue Tek®, Miles Laboratory, Naperville, IL, USA) (4583) on a cardboard and frozen as described below or fixed by immersion overnight at 4 °C in a solution of 4% formaldehyde in 0.1 M phosphate buffer (pH 7.0). These latter muscle samples were then carefully washed in Tyrode’s solution (10% sucrose) at 4 °C overnight and mounted as described above. Both unfixed and fixed samples were then frozen in propane chilled with liquid nitrogen and stored at −80 °C.

### Sectioning and staining

The tissue samples were sliced in 5–8 μm thick sections with a cryostat. The sections were mounted on glass precoated with chrome-alum gelatin.

For *visualization of morphology* of the samples, staining with Haematoxylin & Eosin was performed. Sections from all groups were hereby stained. The sections were defrosted, and then stained in Harris Haematoxylin solution for 2 min. Then the sections were rinsed in distilled water and dipped three times in acetic acid (0.1%). Thereafter the sections were put into hot water for 5 min followed by contrast staining with Eosin (10 ml eosin +90 ml 70% alcohol) for 1 min. At the end, the sections were dehydrated by dipping three times into alcohol. The sections were mounted with Pertex Mounting Medium (art nr. 00840) (Histolab Products, Askim, Sweden).

#### Single immunostainings

For detection of TNFR1 and TNFR2 at protein level immunohistochemistry was performed on sections of chemically fixed tissue. The sections were defrosted and some of them were then put in potassium permanganate (KMnO_4_) for 2 min, according to established procedures [10, 31]. Other sections were processed without KMnO_4_ treatment. The glasses were washed in 0.01 M phosphate buffer saline (PBS) 5 min × 3. Incubation for 20 min in Triton X-100 (T8787, Sigma) in 0.01 M PBS proceeded, followed by washing in PBS 5 min × 3. A following incubation in 5% normal donkey serum (017–000-121, Jackson ImmunoResearch Laboratories, Inc., PA, USA) in PBS, for 15 min, was performed. Then the sections were incubated with primary antibody diluted in PBS for 60 min in 37 °C. Two different primary TNF receptor antibodies were utilized, namely antibodies towards TNFR1 and TNFR2. The antibodies were diluted in PBS. Both antibodies were goat polyclonal IgG antibodies. The TNFR1 antibody was raised against the C-terminus peptide mapping of mouse TNFR1 (Santa Cruz, sc-1070) (dilution used: 1:100). The TNFR2 antibody had been raised against the peptide mapping at the C-terminus of mouse TNFR2 (Santa Cruz, sc-1074) (1:100).

The sections where then washed in PBS 5 min  × 3. Another incubation in normal donkey serum for 15 min followed. The sections were thereafter incubated for 30 min in 37 °C with secondary antibody which correspond to Fluorescein IsoThiocyanate-conjugated (FITC) Donkey Anti Goat (DAG) IgG (705–095-147, Jackson ImmunoResearch Laboratories, Inc., PA, USA), and then diluted in 0.01 M PBS. One last rinsing in PBS for 5 min × 3 was performed and then the sections were mounted with Vectashield Antifade Mounting Medium (H-1000, Vector Laboratories, Inc. Burlingame, CA 94010, USA).

The stainings were accomplished with control stainings including staining when the secondary antibodies were eliminated. Preabsorption of the primary antibody with TNFR1/TNFR2 antigen (100 μg/ml for TNFR1 and 150 μg/ml for TNFR2) (sc-1070P for TNFR1, sc-1074P for TNFR2) in 4 °C overnight was also performed. The next day staining was made using same protocol as described above. Ordinary stainings were made in parallel.

The sections were then examined in microscope (Zeiss Axioskope 2 plus) and pictures were taken by an Olympus DP 70 digital camera.

#### Double immunostaining

For determination of locations of immunoreactions (IR) for TNFR1 and TNFR2, double stainings were done. These were done on unfixed tissue as stainings with antibodies used for these stainings, [mouse monoclonal antibodies against Pax7 (Developmental Studies Hybridoma Bank, University of Iowa, Iowa City, USA), CD31 (M0823, DAKOCytomation, Glostrup, Denmark), βIII-tubulin (T8660, Sigma) and S-100β (S2532, Sigma)] are known to function optimally by using such tissue [32–34]. Different combinations of antibodies were used for the double stainings. Primary antibody (either against TNFR1 or TNFR2, which are goat polyclonal antibodies) was incubated in 4° overnight. Then the sections were incubated with FITC-conjugated secondary antiserum (c.f. above) for 30 min in 37°, and washed with PBS 3 × 5 min. Then followed a new incubation for 60 min in 37° with a different primary antibody (which in all cases corresponded to mouse monoclonal antibody) (c.f. above), use of normal donkey serum as normal serum, washing with PBS 3 × 5 min, after which sections were incubated with Rhodamine Red-X-conjugated (RRX; Jackson ImmunoResearch Laboratories, West Grove, PA, USA) (code 713–295-003) or Alexa fluor 647-conjugated (Invitrogen, CA, USA) (S21374) antiserum. The secondly used primary antibodies conformed to mouse antibodies against Pax7 for detection of satellite cells, CD31 for demarcation of vessel walls (for details, see above), βIII-tubulin (T8660, Sigma-Aldrich, New York, NY, USA) for axons and S-100β (c.f. above) for Schwann cells. Some unfixed sections were also labelled with α-bungarotoxin (alpha-bungarotoxin 594, Molecular Probes, B13423, Invitrogen) for detection of neuromuscular junctions.

Mounting and microscopic evaluation was thereafter made as described above. Some sections were mounted with another medium than described above, namely Vectashield Antifade Mounting Medium with DAPI (H-1500, Vector Laboratories, Burlingame, CA 94010, USA) for marking of nuclei. All mouse monoclonal antibodies used for the double stainings have been used in previous studies and have been successfully tested [33, 35].

The sections which were double-stained were scanned with a fluorescence microscope (Leica DM6000B, Leica Microsystems CMS GmbH, Wetzlar, Germany). Photos were taken with a color CCD camera (Leica DFC490) and a digital high-speed fluorescence CCD camera (Leica DFC360 FX).

*In situ* *hybridization* was performed for TNFR1 mRNA. We hereby selected representative specimens from the 1, 3, 6 week groups (groups 2,5,6) and specimens from the 1 week experiment/injected animals (groups 4a–c), the specimens exhibiting the characteristic appearances that were noted morphologically (in total 11 specimens). Specimens from the control group were also investigated (two animals). The specimens from the experimental animals included specimens from both the experimental and contralateral (non-experimental) sides. The tissues were cut in 10 μm thick fresh cryosections by a cryostat (with a knife washed in 70% EtOH in DEPC-H_2_O) and mounted onto Super Frost Plus slides (nr.041200, Menzel Gläser, Braunschweig, Germany). The procedures were made according to an established protocol [10, 36]. The sequence of the anti-sense probe used was TCCTCGATGTCCTCCAGGCAGCCCAGCAGGTCCATGTCGCGGAGCACG. A corresponding sense DIG-hyperlabeled ssDNA probe was used as a negative control. A β-actin antisense probe (GD-5000-OP) was utilized as a positive control, and was compared to a β-actin sense probe (Gene Detect, New Zealand). The dilution was 50 ng in 15 μl hybridization solution. An alkaline phosphatase (AP)-labelled anti-D16 antibody (Roche Germany, 11,093,274,910) was used for detection. The sections were finally mounted in Pertex mounting medium. For further details of the procedures, see [8, 10].

#### TNFR1 mRNA in relation to desmin/NK-1R

To analyze the relation between muscle fibers expressing TNFR1 mRNA and fiber reorganization/regeneration [10], parallel sections to sections processed for TNFR1 mRNA were processed with immunohistochemistry, via using double staining for desmin (MO760, Dako Cytomation, Glostrup, Denmark) and NK-1R (Sc5220, Santa Cruz Biotechnology, Dallas, TX, USA) on unfixed tissue. Concerning staining for the NK-1R antibody, the procedures conformed to the procedures described above for labeling for TNFR1/TNFR2. That included the use of the same secondary antibody and the same normal serum as described for this labeling. After the staining for NK-1R the staining for desmin followed. As normal serum, rabbit normal serum was used and the secondary antibody conformed to anti-mouse immunoglobulins/TRITC (R0276) (Dako, Denmark). The secondary antibody used in this case was used at a dilution of 1:40. Mounting was made in Vectashield Antifade Mounting Medium (H-1000) or Antifade Mounting Medium with DAPI (H-1500) (Vector Laboratories, Burlingame, USA) in order to identify nuclei.

The NK-1R antibody is produced in goats (sc-5220, Santa Cruz) and is an affinity purified polyclonal antibody raised against a peptide mapping within an internal region of NK-1R of human origin. It was used at a dilution of 1:100 in 0.1% in PBS. The antibody against desmin is a mouse monoclonal antibody and is by the supplier reported to be specific for desmin and to not show reactivity with other types of intermediate filaments. It was used at a dilution of 1:100 in PBS with BSA. Both the NK-1R [10, 32] and the desmin [37] antibodies have been previously characterized and tested.

## Results

### Morphology

**Fig. 1:**
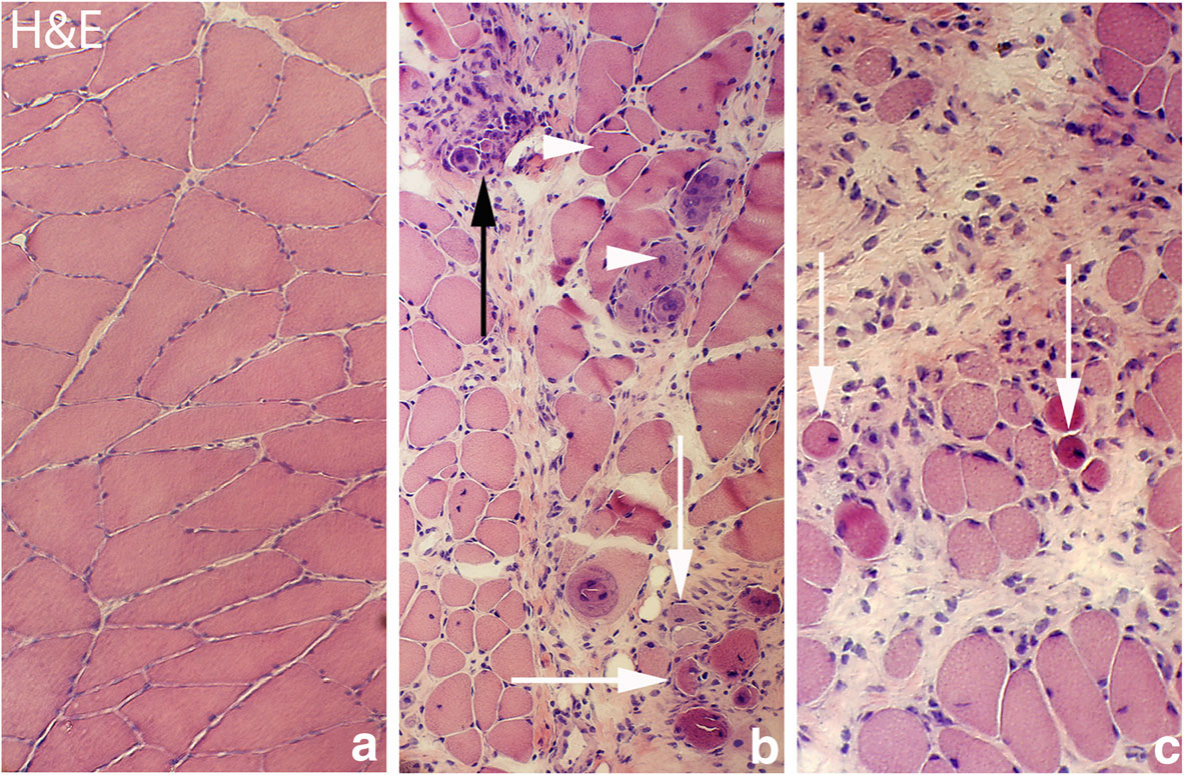
Figure demonstrating hematoxylin & eosin staining (H&E) of muscle tissue. **a** is from the group of the non-experimental animals (control group), representing normal muscle tissue with tightly packed muscle fibers. **b** and **c** show muscle tissue from experimental animal (6 week group, contralateral side) (lower magnification in [**b**] than in [**a**] and [**c**]). In (**b**) and (**c**) there is an abundance of connective tissue with infiltrating white blood cells and abnormally looking muscle fibers that were not seen in the non-experimental group (a myositis situation). Note the marked variability in muscle fiber sizes in (**b**) and (**c**). Black arrow at the place of a necrotic muscle fiber for which the contour is lost and white arrows at muscle fibers that are small and that to varying extents show a basophilic appearance. Fibers with internal nuclei are seen (**b**) (white arrowheads). Orig. magnif. ×300 (**a**, **c**), ×200 (**b**)

The muscles of the control group showed a normal morphology. There were no inflammatory infiltrates (Fig. 1a). In the 1-week group, without injection treatment (group 2), the morphology was still rather normal. Occasionally, partly abnormally looking muscle fibers were seen. In the 3-week group (group 5), streaks of connective tissue with white blood cells (small myositis areas) were seen in the muscle tissue in some of the samples of both experimental and non-experimental sides. However, the majority of the muscle fibers showed a normal appearance. In the 6 week group (group 6), there was a marked change in muscle morphology in comparison to rabbits in the control group (Fig. 1a-c). The changes were seen for both experimental and non-experimental sides. These changes included an increase in connective tissue spaces, occurrence of a large amount of white blood cells within these spaces and presence of a large number of abnormal muscle fibers, as in a myositis situation. A similar pattern was seen for the 1 week group treated with injections of substances (group 4a–c). Not all areas were nevertheless influenced; there were still parts of muscle tissue that had a normal appearance (Fig. 1b, c). In the myositis areas, some of the muscle fibers were invaded by white blood cells (Fig. 1b). These are interpreted as being necrotic muscle fibers. A lot of other abnormally appearing muscle fibers were also seen but that not were invaded by white blood cells. That included muscle fibers that were small and that sometimes could be seen to have a basophilic appearance, as well as muscle fibers that had internal nuclei (Fig. 1b, c) and that contained vacuoles. These types of fibers are further on referred to as “abnormal muscle fibers”.

These morphologic observations conform to those made in previous studies on the experimental model [8, 10].

### In situ hybridization – TNFR1

**Fig. 2:**
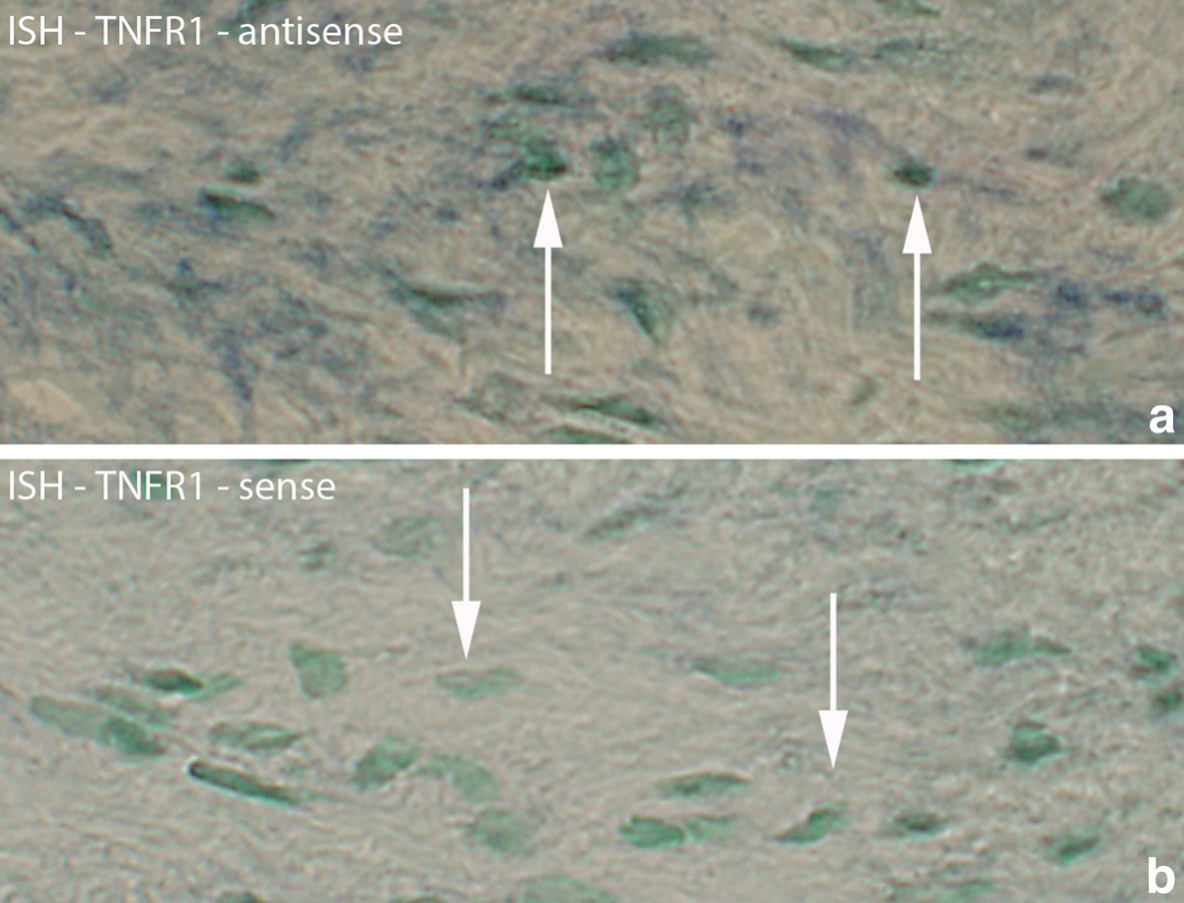
Region from myositis area with fibroblasts processed for in situ hybridization (ISH) for detection of TNFR1 mRNA (**a**). **b** is from a parallel section processed with the corresponding sense probe and working as a control. This sample is collected from experimental animal (3 week group, contralateral side). Arrows at reactive (**a**) and non-reactive (**b**) fibroblasts. Orig. magnif. ×500

Parts of the fibroblasts in the connective tissue spaces of myositis areas were found to express TNFR1 mRNA (Fig. 2). Labeled fibroblasts were found at both experimental and non-experimental sides in the 1 week injected (groups 4a–c), and 3 and 6 week (groups 5,6) groups. There were no fibroblasts expressing TNFR1 mRNA in the 1 week group (group 2) and the control group (data not shown).

**Fig. 3:**
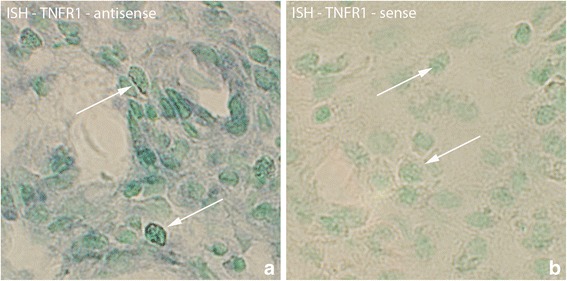
Sections of specimen collected from experimental animal (3 week group, experimental side) processed with ISH for detection of TNFR1 mRNA in white blood cells of myositis area (**a**). TNFR1 mRNA is present in white blood cells (arrows). The parallel section (**b**) is a control section (sense control); arrows at non-reactive cells. Orig. magnif. ×500

TNFR1 mRNA was also found in white blood cells located in the connective tissue of the myositis areas (Fig. 3). Such white blood cells existed in the 1 week injected (groups 4a–c) and 3 and 6 week groups (groups 5,6) bilaterally, but not in the other groups.

**Fig. 4:**
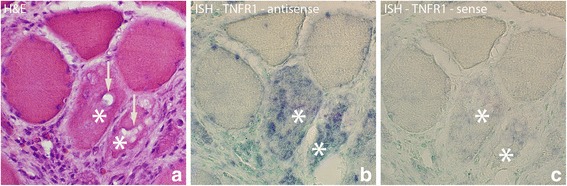
Parallel sections of a group of muscle fibers from 1 week group (experimental side) which are investigated with H&E, ISH antisense and sense TNFR1 mRNA probes. There are two muscle fibers (marked with *) that are different from the others surrounding them, and in which TNFR1 mRNA is present, exhibiting a diffuse but localized pattern (**b**). Note the presence of vacuoles (arrows, **a**) in these muscle fibers. Note also the frequently occurring white blood cells in the connective tissue outside the muscle fibers. That includes eosinophils (red coloured) (**a**). There is no specific reaction in the control (**c**). Orig. magnif. ×300

TNFR1 mRNA expression was also seen to exhibit a diffuse but localized pattern in some of the abnormal muscle fibers (Fig. 4). This type of expression was found bilaterally in the injected groups (groups 4a–c) as well as in the 3 and 6 week groups (groups 5,6), being especially prominent in the 6 week group. Occasionally such an expression was also seen in samples of the 1 week group (group 2) (Fig. 4).

**Fig. 5:**
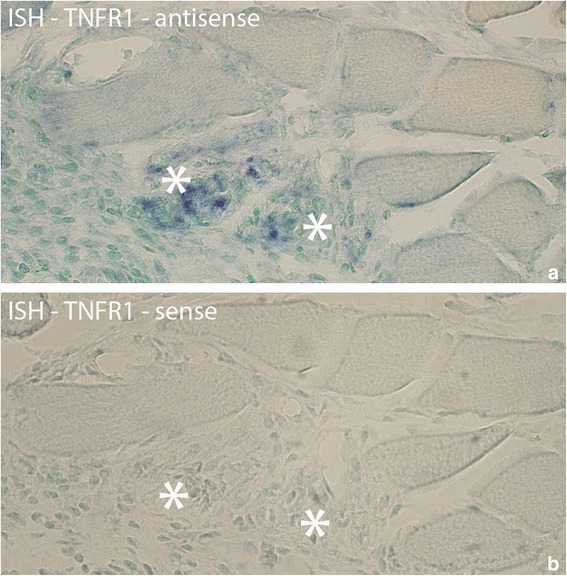
Sections from experimental animal (3 week group, experimental side) processed with ISH; antisense (**a**) and sense (**b**) probes. **a** shows that there are TNFR1 mRNA reactions in two muscle fibers (asterisks) that are very poorly outlined. **b** is a control (use of sense probe). In (**b**), as well as in (**a**), it can be noted that there is cellular infiltration in the two muscle fibers, especially the one to the right, suggesting that they are necrotic fibers. Both necrotic fibers are very much destroyed. Such an infiltration of cells was not seen in the muscle fibers expressing TNFR1 mRNA in Fig. 4. Note that the other muscle fibers seen in the fig appear normal. Orig. magnif. ×300

There were also TNFR1 mRNA reactions in the necrotic muscle fibers (Fig. 5). The reaction resided in the white blood cells that had invaded the fibers.

### TNFR1 mRNA in relation to desmin and NK-1R

In our previous paper [10], it was noted that abnormal non-necrotic muscle fibers that were interpreted to possibly be in a regenerative stage showed TNF-alpha mRNA, a marked non-striated desmin immunoreaction pattern and NK-1R IR. These features suggested that the fibers presumably were in a regenerative stage [10].

**Fig. 6:**
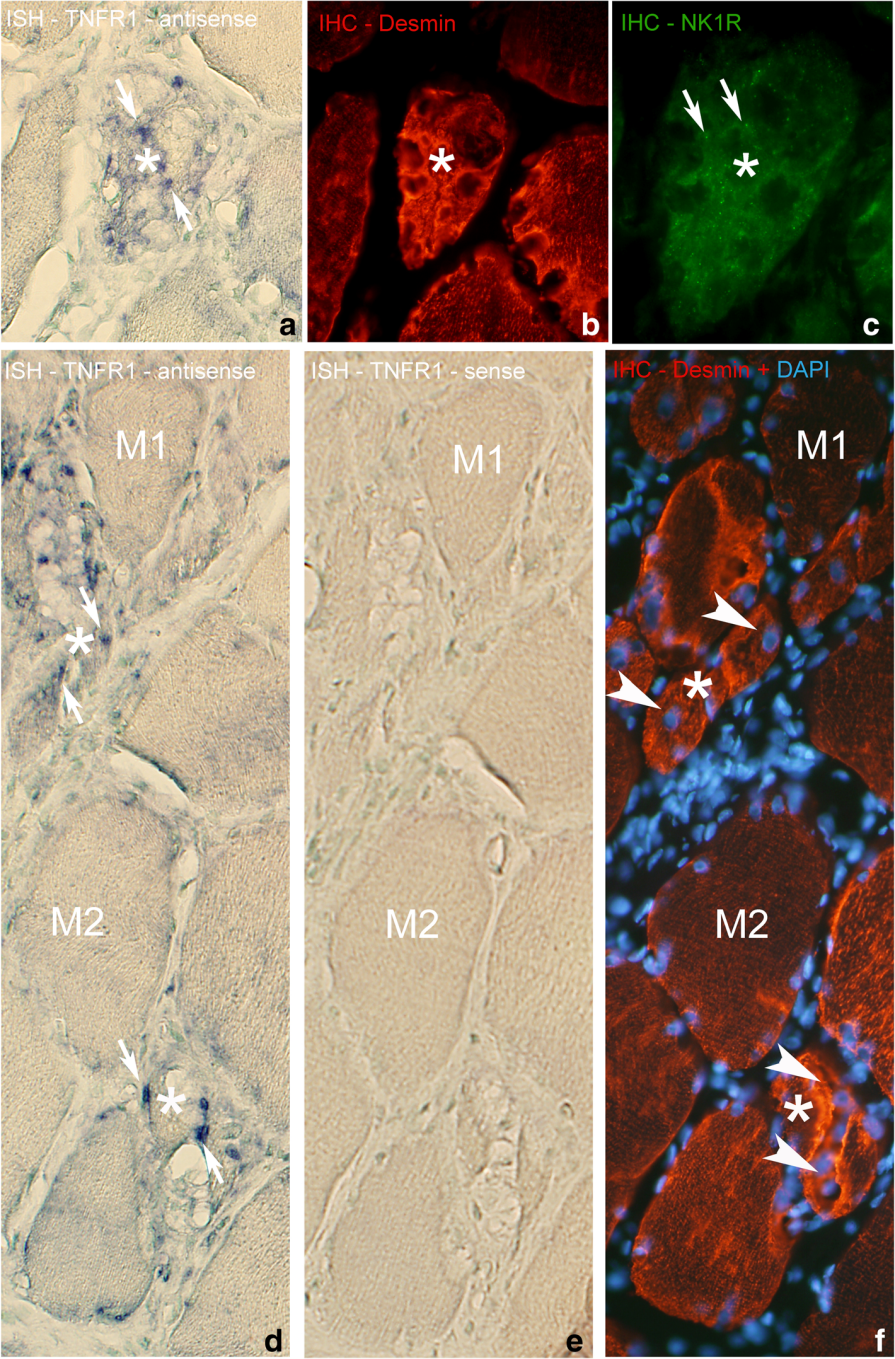
**a**-**c** Parallel sections of muscle fibers, processed with ISH for TNFR1 mRNA (**a**) and immunohistochemistry (IHC) with antibodies towards desmin (**b**) and NK-1R (**c**). Please note that (**c**) is in higher magnification than (**a**) and (**b**). **a** shows that there is an abnormal muscle fiber expressing TNFR1 mRNA in the middle, in which in (**b**) a high expression of desmin is seen. In (**c**), there is an existence of point-like NK-1R immunoreactions in this muscle fiber (arrows). The muscle fiber is not filled with fine cells of the type seen in Fig. 5. Also the muscle fiber to the right is to some extent abnormal, partly showing high expression of desmin (**b**). The muscle fiber below in (**b**) shows a more normal, striated, desmin pattern. Sample from experimental animal (6 week group, contralateral side). Asterisk at corresponding location. Orig. magnif. ×300. **d**-**f** Sections of specimen of 6 week experimental group (contralateral side) processed with antisense and sense TNFR1 mRNA probes (**d**, **e**) and for desmin IHC (**f**) (in **f**, processing for DAPI was performed). Some of the muscle fibers appear abnormal, several of them being small in size (asterisks), whilst others appear normal (M1, M2). Note the presence of TNFR1 mRNA (white arrows, **d**) in the abnormally appearing small muscle fibers but not in those with normal morphology. Note also the presence of internal nuclei in the abnormal muscle fibers (white arrowheads, **f**). As in **a**-**c** the abnormal muscle fibers are not filled with fine cells of the type seen in Fig. 5. There are no specific reactions in (**e**). Orig. magnif. ×300

Based on these findings, parallel sections to those processed for TNFR1 mRNA were analyzed with immunohistochemistry for desmin and NK-1R. The stainings showed that there was a marked and generalized desmin IR and a fine point-like NK-1R IR in the muscle fibers expressing TNFR1 mRNA (Fig. 6). Muscle fibers showing a normal morphology, exhibited a striated desmin immunoreaction pattern characteristically seen in normal skeletal muscle, and a non-existence of NK-1R immunoreactions.

### Immunohistochemistry – TNFR1 and TNFR2

#### Fibroblasts

**Fig. 7:**
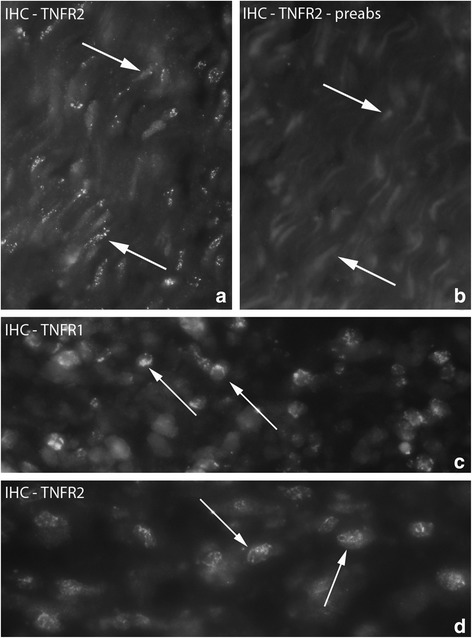
**a**, **b** Figure showing fibroblasts of myositis area. Sample from experimental animal (6 week group, experimental side). The section in (**a**) is processed for IHC (TNFR2). Fibroblasts exhibit TNFR2 IR (arrows). **b** is processed with preabsorbed TNFR2 antibody and works as a control (arrows at non-immunoreactive fibroblasts). Orig. magnif. ×500. **c**, **d**. Figure showing presence of TNFR1 and TNFR2 IR in white blood cells. Samples from 1 week experimental animal; experimental side (injected; group 4a). Arrows at white blood cells with reaction for receptors. Orig. magnif. ×500

Fibroblasts in the connective tissue spaces of the myositis areas of 1 week injected (groups 4a–c), the 3 week group and especially the 6 week group (groups 5,6) expressed TNFR2 IR. The TNFR2 IR was most clearly seen for the 6 week group (group 6) (Fig. 7a) and was here noted bilaterally. TNFR2 IR was not seen for fibroblasts in the control group and the 1 week group not given injections (group 2) (data not shown). Only a few fibroblasts with TNFR1 protein were seen in the 6 week group (group 6). TNFR1 IR was not seen in fibroblasts in any other groups.

#### White blood cells

Both TNF receptors were found to be expressed in white blood cells located in the connective tissue of myositis areas on both sides (Fig. 7c, d). TNFR2 IR was seen in most of the white blood cells. Although TNFR1 mRNA could be noted in the white blood cells (c.f. above), TNFR1 IR was much less frequently seen than TNFR2 IR in the white blood cells.

#### Muscle fibers

**Fig. 8:**
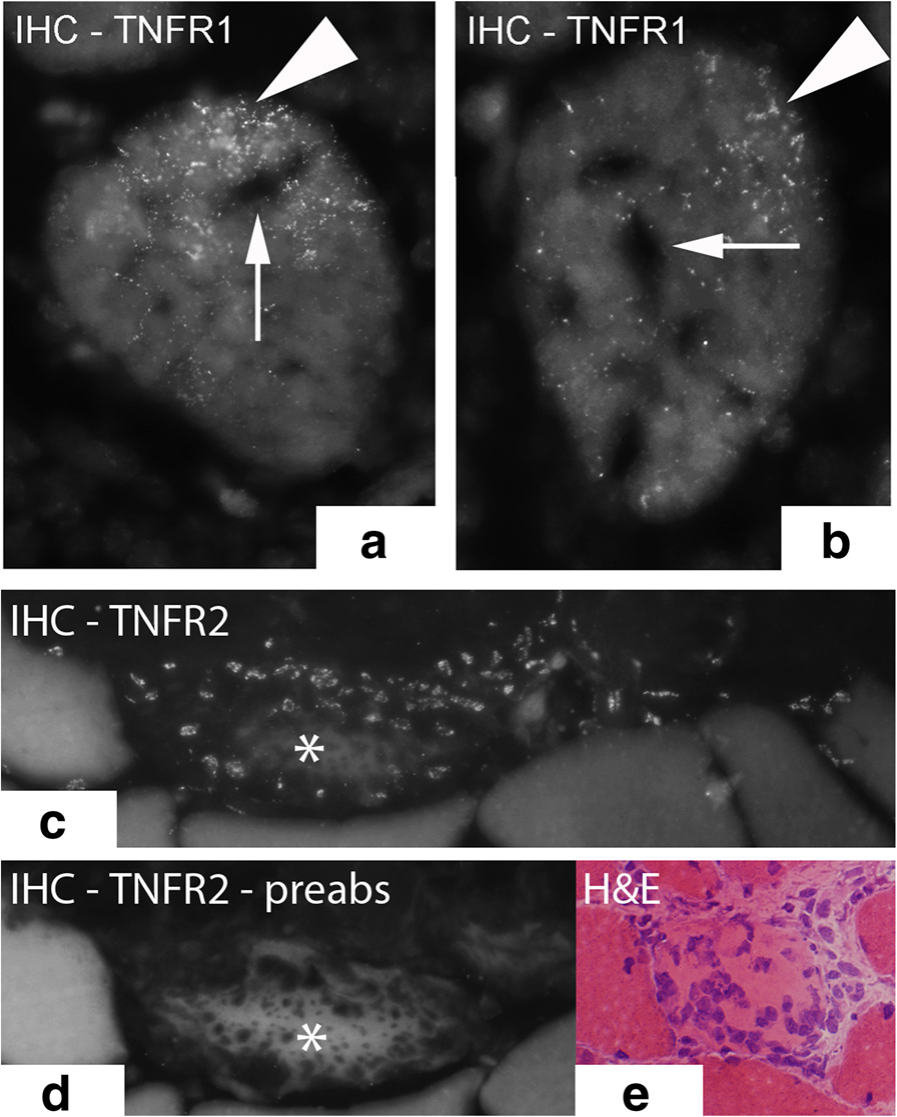
TNFR1 IR expression in the cytoplasm of muscle fibers, the reactions showing a finely spread and dot-like pattern (**a**, **b**) (arrowheads). Sample from 6 week group, contralateral side (arrows at unstained areas). **c** and **d** show a much destroyed necrotic muscle fiber (asterisks) partly being infiltrated by white blood cells TNFR2 IR (**c**) preabsorption with TNFR2 antigen in (**d**). The white blood cells express TNFR2 IR. Sample from experimental animal (3 week group, contralateral side). **e** shows another example of muscle fiber being infiltrated by white blood cells and being stained with H&E in a specimen from experimental animal (6 week group, contralateral side). Orig.magnif. ×500 (**a**), ×300 (**c**-**e**)

TNFR1 IR was spread in a dot-like fashion in the cytoplasm in some of the abnormal non-necrotic muscle fibers of myositis areas (Fig. 8a, b). There was no difference between experimental and non-experimental sides in this respect. This type of immunoreaction for muscle fibers was the most distinct in the 6 week group (group 6). There was no TNFR1 expression in the muscle fibers of the control group (data not shown) and an immunoreaction of this type in muscle fibers was not seen in any group concerning TNFR2.

 TNFR2 IR was also seen in necrotic muscle fibers. The pattern of TNFR2 IR was different from that seen for TNFR1 in abnormal muscle fibers described above. The TNFR2 IR in the necrotic muscle fibers was in the form of immunoexpression for invaded white blood cells (Fig. 8c, d).

**Fig. 9:**
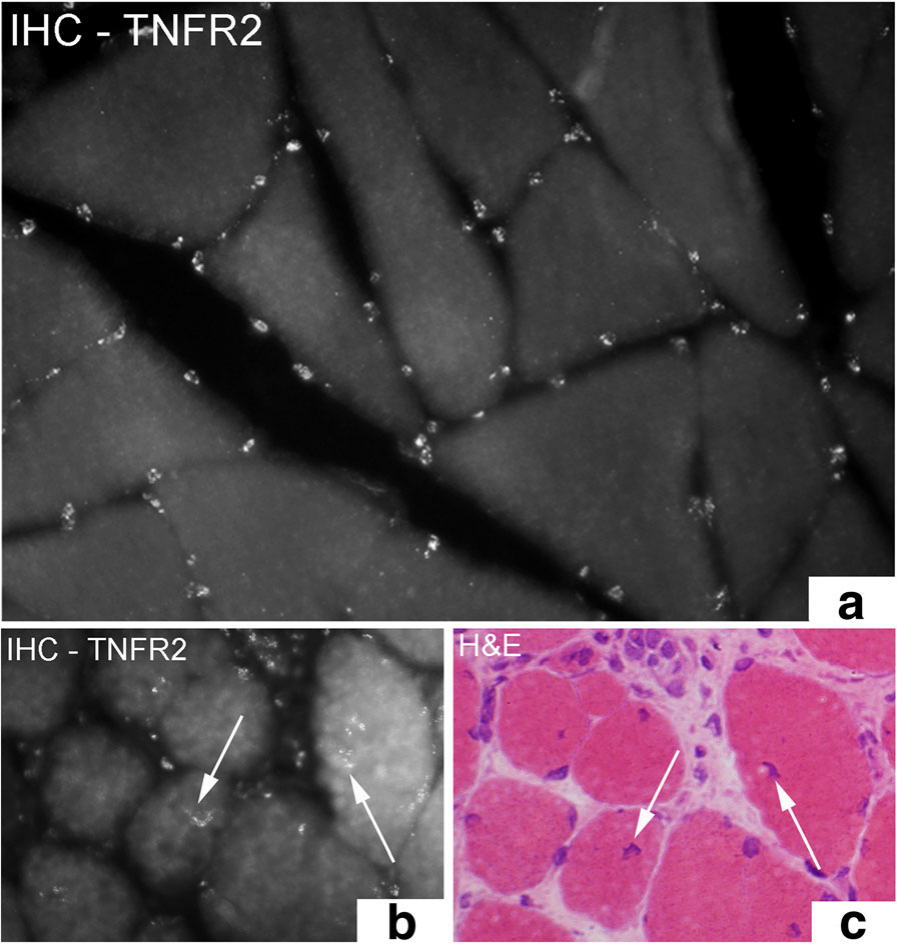
Muscle fibers stained with IHC for the detection of TNFR2 in (**a**) and (**b**). In (**a**), there is localized TNFR2 immunoreactions in the outer parts of the muscle fibers while in (**b**), there is also TNFR2 immunoreactions localized to the middle of muscle fibers (arrows). **c** shows a parallel section to (**b**) stained with H&E which displays that the immunoreactive structures represent internal nuclei. The specimens are from 6 week experimental group; contralateral side. Orig. magnif. ×300 (**a**-**c**)

**Fig. 10:**
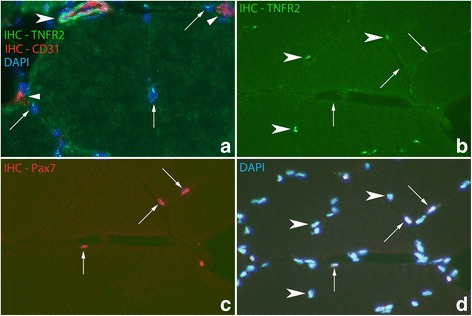
**a** Double staining (TNFR2/CD31) in section from specimen of 6 week experimental group; contralateral side. In the top, a blood vessel is seen being located outside a muscle fiber, showing CD31 IR (red colour) in the internal (intima) part and TNFR2 IR (green colour) in the outer part (large arrowhead). Arrows at nuclei located in peripheral parts of muscle fibers (marked with DAPI) in association with which TNFR2 IR is seen (appearing as fine dot-like whitish reactions). Fine CD31 immunoreactions (red colour) (small arrowheads), representing capillaries, and in which there are no TNFR2 immunoreactions, are also seen. There is a high auto fluorescence background reaction in the cytoplasm of the muscle fiber. Orig. magnif. ×300. **b**-**d** Figure showing double staining (IHC) for TNFR2 and the satellite cell marker Pax7. It is also labelled with DAPI. TNFR2 is not expressed in the satellite cells (arrows); there is no co-localization (**b** and **c**) (arrows at Pax7 positive cells). TNFR2 IR is on the other hand seen in some muscle cell nuclei (arrowheads, **b** and **d**). Sample from experimental animal; contralateral side (6 week group). Orig. magnif. ×300

There were also localized TNFR2 immunoreactions in the outer part of the muscle fibers (Fig. 9a). This expression pattern was seen for all groups, including those in the control group. Double stainings with Pax7 and CD31 and labeling with DAPI showed that the localized TNFR2 immunoreactive structures in the outer parts of the muscle fibers were not satellite cells nor capillaries but muscle fiber nuclei (Fig. 10). Localized immunoreactions in the outer parts of muscle fibers were not seen concerning TNFR1.

In the experimental animals, localized TNFR2 immunoreactions were also seen in internal nuclei of the muscle fibers (Fig. 9b). That was never the case concerning TNFR1.

#### Blood vessel walls

**Fig. 11:**
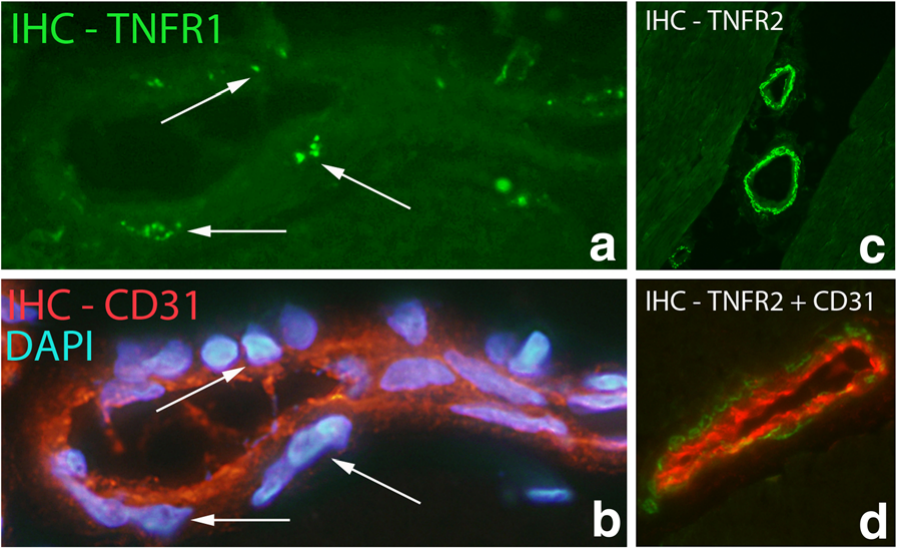
**a**, **b** The figures represent double staining (IHC) of a vessel, with antibodies towards TNFR1 and CD31. The section in (**b**) is also labelled with DAPI. TNFR1 is expressed in cell nuclei of the smooth muscle (arrows) but not in the endothelium of the vessel. Sample from experimental animal; experimental side (6 week group). Orig. magnif. ×500. **c**, **d**. Staining with antibody against TNFR2 (green colour) (**c**). **d** represents double staining; staining with antibodies against TNFR2 (green colour) and CD31 (red colour). TNFR2 is seen in the outer (smooth muscle) part of blood vessel walls in both (**c**) and (**d**) but not in the inner (intima) parts. There is expression of CD31 in the inner layer (**d**). Specimens from 6 week experimental group, contralateral side. Orig. magnif. ×300 (**c**), ×500 (**d**)

TNFR2 IR was seen in blood vessel walls in all groups, but the reaction was most prominent for the blood vessel walls in the experimental groups (Fig. 10a). There was no TNFR2 IR in walls of capillaries (Fig. 10a). The TNFR2 IR was positioned just outside the CD 31 IR layer, i.e. in the smooth muscle layer (Fig. 10a, 11c, d). TNFR1 IR was only sometimes seen in blood vessel walls (Fig. 11a, b) and was then restricted to the nuclei in the smooth muscle layer of the vessel walls.

#### Nerve fascicles

**Fig. 12:**
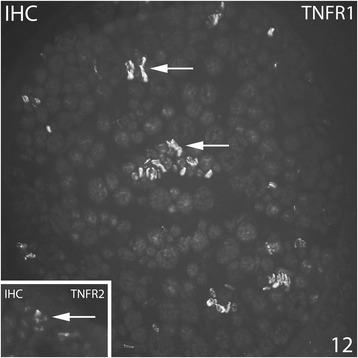
Specimens from 6 week experimental group (contralateral side). Large part of a nerve fascicle is shown (small part of a nerve fascicle in inset). IHC staining for TNFR1 and TNFR2. Immunoreactions are seen for both receptors (arrows). Orig. magnif. ×300

**Fig. 13:**
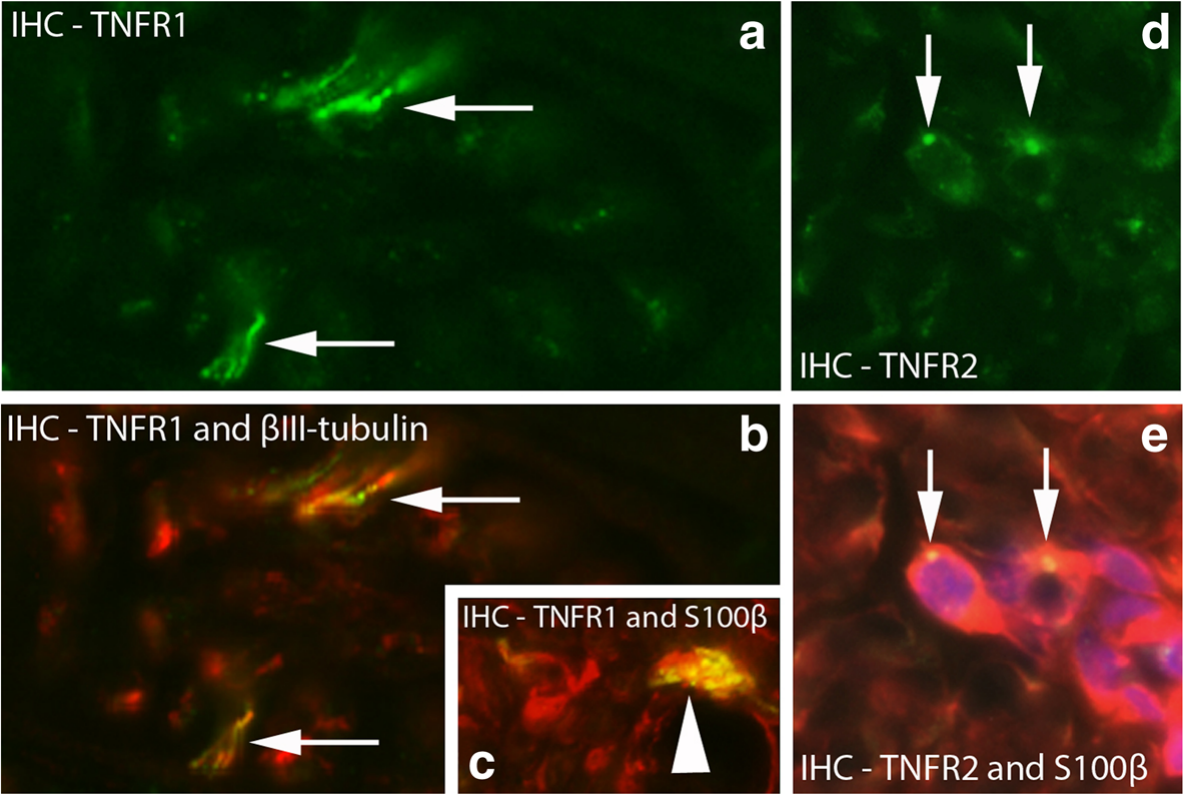
**a**, **b** Double staining (IHC) of nerve fascicle with antibodies towards TNFR1 (green colour) (**a**) and βIII-tubulin (merged reactions in **b**). There is co-localization between TNFR1 and βIII-tubulin IR in nerve profiles in (**b**) (arrows). Sample from experimental group (6 week group; contralateral side). Orig. magnif. ×500. **c**. Double staining for TNFR1/Schwann cell marker S-100β: Small part of a nerve fascicle is shown. There are TNFR1 reactions (green-yellowish) in one of the Schwann cells (red) (arrowhead). Sample from experimental group (6 week group; experimental side). Orig. magnif. ×500. **d**, **e**. Double staining TNFR2/S-100β [TNFR2 reaction in green in (**d**), merged reactions in (**e**); S-100β in red)]. There is TNFR2 IR in S-100β stained cells, i.e. Schwann cells. Arrows at the TNFR2 immunoreactive spots. Sample from 6 week group, contralateral side. Orig. magnif. ×500

TNFR1 and TNFR2 immunoreactivities were only very occasionally seen in nerve fascicles in the control group as well as in the 1 week group (group 2). In the 1 week injected and the 3 and 6 week groups (groups 4–6), expressions for both TNF receptors were more regularly seen in the nerve fascicles and the intensities of immunoreactions were stronger (Fig. 12). That was the case for the experimental as well as the non-experimental sides. Double stainings with TNF receptors, S-100β and βIII-tubulin showed that TNFR1 IR was present in Schwann cells and axons, whilst TNFR2 IR was only seen in the Schwann cells (Fig. 13).

#### Neuromuscular junctions (NMJ)

**Fig. 14:**
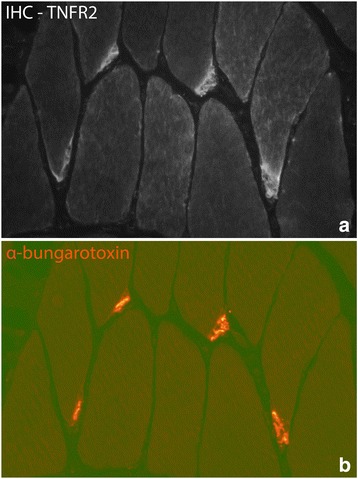
Parallel stainings (IHC) towards TNFR2 (**a**) and the NMJ marker α-bungarotoxin (**b**). The figures demonstrate the presence of TNFR2 in the NMJ. Sample from experimental animal (6 week group, experimental side). Orig. magnif. ×300

As marker for NMJ, staining for α-bungarotoxin was used. It was found that TNFR2 IR was confined to the NMJ for all the groups (Fig. 14). TNFR1 was never seen for NMJ.

#### Quantifications

The degrees of labellings for TNFR1 IR and TNFR2 IR concerning muscle fibers, white blood cells, nerve fascicles and the neuromuscular junctions for the various groups were calculated. Based on the labellings, semi quantitative estimations were made (see Table 2).

**Table 2:** Table showing semi quantitatively the magnitude of expressions for TNFR1 and TNFR2 immunoreactions (IR)

	Animals showing normal morphology TNF Receptor type 1	Animals showing normal morphology TNF Receptor type 2	Animals expressing myositis TNF Receptor type 1	Animals showing myositis TNF Receptor type 2
Abnormal muscle fibers	-	-	++	-
Necrotic muscle fibers with invading cells	-	-	-	++
Muscle nuclei	-	+++	-	+++
White blood cells	-	-	+	+++
NMJ	-	+++	-	+++
Nerve fascicles	(+)	(+)	++	++

– (no reaction), (+) (very limited reaction, + (moderate reaction), ++ (marked reaction), +++ (very marked/regularly occurring reaction). The animals for which a clear myositis was seen (related to most animals from groups 4 and 6 and sometimes those from group 5) and for which a normal muscle morphology occurred (related to animals from groups 1–3 and occasional animals from group 5)

## Discussion

The present study shows that there is a marked upregulation of TNFR1 and TNFR2 IR in the rabbit muscle tissue in animals subjected to marked overuse. This includes expressions in the muscle fibers, the nerve fascicles, the blood vessel walls, the white blood cells and the fibroblasts. There were nevertheless differences between the reaction patterns for the two receptors. In contrast, very restricted reactions were observed in the control animals.

In regards to the morphology, it was evident that a myositis had developed, especially in the 3 and 6 week groups and the 1 week group animals that were given pro-inflammatory substance (groups 3–6). This is in accordance with the observations on the model we have previously made [8, 10, 38]. Certain muscle fibers in the myositis areas were necrotic whilst others showed an abnormal morphology in other ways. The latter muscle fibers contained varying extents of internal nuclei and vacuoles, were sometimes basophilic and were of varying sizes, often being small. They are in Results referred to as abnormal muscle fibers.

### Expressions in muscle fibers

Some of the abnormal muscle fibers that were not necrotic showed frequently a marked non-striated desmin immunoreaction pattern. This feature suggests that they are in a reparative/regenerative stage. It is known that there initially is a loss of desmin in response to muscle injury but that there later on is a tremendous quantitative increase in desmin content, interpreted to be related to protective effects [39]. Overexpression of desmin and a non-striated immunoreaction pattern for this protein is actually reported for regenerative phases of the muscle fibers [40]. Occurrence of a strong and generalized reaction for desmin in parallel with features such as small fiber sizes, presence of internal nuclei and a basophilic cytoplasm have also previously been interpreted as markers for possibly regenerating muscle fibers [41].

TNFR1 mRNA was observed as diffuse but localized reactions in abnormal muscle fibers exhibiting a strong and generalized desmin IR. Immunohistochemically, TNFR1 was seen to be widespread in a dot-like pattern within abnormal muscle fibers. Such an immunoreaction pattern was never observed for TNFR2. The functional importance of the observations could be that TNFR1 is related to reparative/regenerative features in muscle fibers in myositis areas. This implies that these muscle fibers, although they are present in myositis areas, possibly can be repaired and not become necrotic.

An interesting fact is that the muscle fibers showing a strong and non-striated desmin IR and a TNFR1 mRNA reaction exhibit NK-1R IR. This is not surprising as it is known that there is an established interrelationship between TNF-alpha and substance P (SP) for which NK-1R is the main receptor. Thus, the production of TNF-alpha from inflammatory cells can be activated via stimulation of SP [42] and blocking of the NK-1R leads to a marked reduction of TNF-alpha production in the joint cavity as seen in studies on experimental inflammation for the temporomandibular joint [43]. Furthermore, in studies on chronic gastritis, it has been found that an increased SP activation can lead to an increased TNF-alpha production [44]. Thus, it may be speculated that in a stressful and damaging process for the muscle tissue, a NK-1R activation occurs in parallel with an activation of TNF-alpha in a reparative/regeneration process for the muscle fibers.

TNFR2 IR was regularly seen to be localized in outer parts of the muscle fibers for all groups but was also observed in the internal nuclei in the center of some muscle fibers for experimental animals. TNFR1 IR did not demonstrate these features. Double stainings and stainings with DAPI revealed that the reactions localized at the outer parts of the muscle fibers were related to reactions for internal nuclei, whilst satellite cells as well as capillaries were non-reactive. The findings of TNFR2 IR but not TNFR1 IR for the internal nuclei can be related to a potential function of TNF-alpha to promote repair and proliferation of the muscle fiber population via TNFR2.

The observations do overall indicate that TNF-alpha is involved in the changes that occur for muscle fibers in the case of an overuse situation. According to what is discussed above, attempts for reparation can occur via both TNFR1 and TNFR2. Earlier studies of our research group on the rabbit experiment model [10] showed that TNF-alpha mRNA is found within both necrotic/degenerating muscle fibers, the reactions being observable in infiltrating white blood cells, and in presumably regenerating muscle fibers. This means that there are cells producing TNF-alpha in relation to muscle fibers [8] but that there also is a basis for TNF-alpha associated effects for the fibers via TNFR1 and TNFR2 pathways.

It is possible that TNF-alpha derived from infiltrated white blood cells, which are likely to be macrophages [45], may play an important role in the development of the myositis, as was suggested for TNF-alpha in the myositis process that was triggered by alphavirus infection in mice [46]. Actually, late-activated macrophages are suggested to contribute to a myositis pathology [47]. On the other hand, TNF-alpha can also influence a simultaneously developing reparation/regeneration process via white blood cell produced TNF-alpha. According to Tidball and Villalta [18], infiltrating macrophages can thus play a major role in promoting growth and regeneration following muscle damage.

### Expressions in nerve structures

An interesting finding is the presence of the TNF receptors in nerve structures. This observation was nevertheless very rarely made for the control and 1 week group (group 2), but with longer experimental period or via the proinflammatory substance injections for 1 week animals (groups 3–6), the expression of receptors in nerve structures was more clearly seen. This finding suggests that effects on nerves gradually increase in response to the experiment condition. While expression for TNF-alpha and its receptors have been very seldom noted for nerves in previous studies on various organs, expressions for TNF-alpha, TNFR1 and TNFR2 have been previously found in nerve trunks in relation to peripheral nerve injury [48]. Furthermore, TNFR1 and TNFR2 are expressed in numerous nociceptors in inflammation-related pain as well [49]. It has also been demonstrated that anti-TNF-alpha treatment has a rapid effect on the nervous system. Via performing fMRI, Hess and colleagues [50] thus found that in response to this treatment, rheumatoid arthritis patients experienced rapid pain relief.

The results of double stainings showed that TNFR1 IR was present in both axons and Schwann cells, whilst TNFR2 was only seen in Schwann cells. These findings are in agreement with findings of TNFR1 expression in neurons and certain of the glia cells and TNFR2 in several of the glia cells but not in neurons in a study on experimental autoimmune encephalomyelitis [51]. Concerning the findings of TNFR2 IR for Schwann cells it should be noted that TNF-alpha can stimulate Schwann cell proliferation [52]. This is in accordance with the well-known fact that signaling via TNFR2 is related to proliferation signaling and neuroprotection [53, 54].

We noted that TNFR2 IR was regularly detected in the NMJ of all groups of animals. The NMJ did not show TNFR1 in any of the animals. Such an observation has to the best of our knowledge never before been made. The observation shows that TNF-alpha effects at the NMJ are effectuated via the TNFR2. Such an interpretation is in accordance with the finding that deletion of TNFR2 impairs motor performance, as seen in study on young and aged mice [55].

### Expressions in blood vessel walls

TNFR1 IR was only sometimes seen in nuclei of the smooth layer of the walls, while TNFR2 IR was regularly seen in the smooth muscle layer of blood vessels, being especially prominent in those of experimental animals. The finding of a difference between reaction patterns for TNFR1 and TNFR2 suggests that the two receptors can have different functions in the blood vessels. In comparison, it is shown that TNFR1 and TNFR2 play different roles in ischemia-triggered angiogenesis, as well as arteriogenesis [56]. TNF alpha is on the whole reported to have an effect on the proliferation of vascular smooth muscle cells [57].

### Findings of a bilaterality

It was observed that changes in TNF receptor expressions for the experimental animals not only occurred for the experimental side but for the contralateral side as well. That included reactions observed for the muscle fibers, the nerve fascicles, the white blood cells and the fibroblasts. Similar findings of a bilateral effect for the overuse model used were noted in regards to the tachykinin system [32]. These findings show that there is an obvious contralateral effect in our overuse muscle model. Contralateral effects after unilateral experiments have also been previously noted. Presumably the first study to demonstrate a contralateral effect was a study by a research group from Yale University in the end of the nineteenth century [58]. They let one of the authors exercise the right hand for ten days but saw a strength increase in not only the right but also the left hand. Since then, many investigators established that there are positive contralateral effects of unilateral training [59]. One condition which is known to demonstrate bilateral effects is Achilles tendinosis. Patients diagnosed with the condition in both legs can get bilateral pain relief after operation on one of the tendons [60].

Bilateral effects related to the TNF alpha system have previously been observed. In experimental studies it was noted that sciatic and spinal nerve ligature resulted in an increased TNFR1 in the neuronal bodies of both ipsi- and contralateral dorsal root ganglia [61]. The neuronal cell bodies were found to bilaterally exhibit an enhanced immunoreaction for TNF-alpha after the sciatic nerve transection [61]. TNF-alpha neutralization with etanercept or infliximab treatment of unilateral antigen-induced arthritis in rats significantly reduced infiltration of macrophages into dorsal root ganglia bilaterally [62]. In experimental studies on a murine model of thermal hyperalgesia it has been shown that unilateral injection of TNF-alpha leads to thermal hyperalgesia in both the inflamed and the contralateral uninjured hind paws [63].

As there was a distinct expression for both TNF receptors in the nerve fascicles bilaterally in experimental animals, it is logical to ask the question whether TNF-alpha can be involved in spreading the myositis process to the contralateral side via the nervous system. This aspect needs to be further investigated.

## Conclusions

It has earlier been shown that musculoskeletal disorders due to overuse in humans lead to elevated levels of inflammatory biomarkers, including TNF-alpha [64]. The present study adds information via showing the expression patterns of the TNF receptors in response to marked muscle overuse. It is shown that there are pronounced TNFR1 and TNFR2 immunoreactions in the soleus muscles of the animals that were exposed to the overuse. This was not the case for the controls. The findings thus suggest that the overuse model leads to a marked involvement of TNF-alpha in the developing myositis process. That was not only related to an involvement in the inflammation process as such but, most interestingly, expression of the receptors was noted for both muscle fibers and nerve fascicles. This demonstrates that in myositis both the muscular system and the nervous system are affected by TNF-alpha. A limitation of the study may be that effects of electrical stimulation was partly a basis for the overuse. Nevertheless, via using the model in the way that was done the features in the developing myositis could be clearly followed.

Whether or not blocking of TNF-alpha effects are worthwhile for skeletal muscle in overuse situations is an interesting aspect which remains for the scientific community to be further investigated. What is known is that blockade of TNF-alpha in exercised dystrophic mdx mice leads to a reduced histological evidence of muscle fiber damage [4]. Interestingly, in the absence of exercise for the dystrophic mdx mice, there was no evidence of a reduction of muscle fiber damage [4]. It is actually suggested that TNF-alpha may be a target for myositis development [65, 66].

As is discussed above, it is possible that effects of TNF-alpha are important in the phase of reparation/regeneration. In our opinion, it can therefore not be excluded that TNF-alpha blocking can have negative effects in the reparation phase after the muscle damage/myositis. It is on the whole obvious that the experience of anti-TNF treatments is very limited for myositis and that more research is needed in order to clarify the effects of this type of treatment in the condition [67].

## Abbreviations

ACE: Angiotensin converting enzyme
CD31: Cluster of Differentiation 31 (or Platelet endothelial cell adhesion molecule)
DAPI: 4′,6-diamidino-2-phenylindole
DIG: Digoxigenin
FITC: Fluorescein isothiocyanate
H&E: Hematoxylin and Eosin
IHC: Immunohistochemistry
IR: Immunoreactions
ISH: In situ hybridization
KMnO_4_: Potassium permanganate
NaCl: Sodium chloride
NF-κB: Nuclear factor kappa-light-chain-enhancer of activated B cells
NK-1R: Neurokinin 1 receptor (same as tachykinin receptor 1 or substance P receptor)
NMJ: Neuromuscular junctions
Pax-7: Paired box protein pax-7
PBS: Phosphate-buffered saline
RRX: Rhodamine red-X
S-100β: S-100 calcium binding protein B
SP: Substance P
TNF-alpha: Tumor necrosis factor alpha
TNFR1: Tumor necrosis factor receptor type 1
TNFR2: Tumor necrosis factor receptor type 2
